# Behavioural Evaluation of a Leash Tension Meter Which Measures Pull Direction and Force during Human–Dog On-Leash Walks

**DOI:** 10.3390/ani10081382

**Published:** 2020-08-10

**Authors:** Hao-Yu Shih, Fillipe Georgiou, Robert A. Curtis, Mandy B. A. Paterson, Clive J. C. Phillips

**Affiliations:** 1 Centre for Animal Welfare and Ethics, University of Queensland, White House Building (8134), Gatton Campus, Gatton, QLD 4343, Australia; mpaterson@rspcaqld.org.au (M.B.A.P.); c.phillips@uq.edu.au (C.J.C.P.); 2 School of Mathematical and Physical Sciences, University of Newcastle, University Drive, Callaghan, NSW 2308, Australia; fillipegeorgiou@hotmail.com; 3 RobacScience Ltd., Wentworth Falls, NSW 2782, Australia; robac@ieee.org; 4 Royal Society for the Prevention of Cruelty to Animals Queensland, Brisbane, QLD 4076, Australia

**Keywords:** dog, leash, tension, human–dog interaction, shelter

## Abstract

**Simple Summary:**

A tense leash when walking a dog is a critical animal welfare issue as it potentially causes damage to a dog’s neck and eyes. This article introduces an innovative canine leash tension meter, for dogs walked on-leash, considering effects of dog age, size–weight, and dogs’ behaviour during walks, to validate the meter. It is confirmed that this device is a robust and valid approach in exploring interactions between dogs and humans when walking on a leash, by real-time measuring the leash tension and differentiating who, dog or human, is pulling the leash.

**Abstract:**

Leash tension forces exerted by dog and handler during walks affect their welfare. We developed a novel ambulatory measurement device using a load cell and a tri-axial accelerometer to record both the tension and direction of forces exerted on the leashes. Data were relayed telemetrically to a laptop for real time viewing and recording. Larger and heavier dogs exerted higher leash tension but had a lower pulling frequency than their smaller and lighter conspecifics. This pattern was observed in the reactional forces of handlers. Young dogs pulled more frequently during walks, which was also mirrored in handlers’ pulling. Well-behaved dogs created lower leash tension, but handlers did not respond with lower forces. This novel method of recording leash tension will facilitate real-time monitoring of the behaviour of dogs and their handlers during walks.

## 1. Introduction

In many countries, dogs are required to be walked on-leash in public areas. Despite increasing emphasis on loose leash heelwork, a tense leash is still common. Lunging or putting too much pressure on a dog’s neck causes damage to the trachea and increases intraocular pressure [1].

Intraocular pressure and neck pressure have been measured with dogs trained to pull on the leash on command, with leash tension measured with a digital force gauge [1]. In addition, collar types were found to influence the pressure distribution on the neck, and all tested collars could potentially injure the dog. In that study, authors used a tension load cell to investigate how different collars affect the pressure on an artificial neck when pulled by different forces [2]. In both studies, fixed forces of pulling were determined in advance [1,2]. In actual walks, leash tension is rarely constant. Recording real-time leash tension could provide a better understanding of the relationship between leash tension and canine, human and environmental variables. Authors could find no research that differentiated between walker and dog leash tension during walks.

In equitation science, rein tension meters have been developed to measure horse rider’s communication with their horses [3,4], based on two load cells relaying forces telemetrically [5]. The load cell used for rein force measurements convert the magnitude of a tension force into a proportional electrical signal which may be recorded or transmitted [4,6,7,8].

If this technique is applied to dogs on leashes, it is also important to distinguish the leash tension contributed by both animal and human handler. A triaxial accelerometer provides quantitative measurements of animal movement in the X, Y, and Z directions including a gravitational component (depending on the device orientation) [9,10]. Combining the measurement of both leash tension by load cell and force direction by acceleration, this paper explores the determination of leash tension contributor to enable the behavioural study of dog and human interaction.

This article introduces the canine leash tension meter for dogs walked on-leash, considering effects of dog age, size–weight, and in-shelter behaviour, to validate the meter. It is hypothesized that dog size–weight is positively correlated with leash tension due to the larger muscle mass in larger and heavier dogs and that human handlers pull the leash harder when walking larger and heavier dogs in order to control them. It is expected that dog age would be negatively correlated with pulling frequency because older dogs are generally calmer [11], and that dogs rated by shelter staff as “well behaved” during the walk would create lower tension on the leash and require less corrections to be performed by handlers.

## 2. Materials and Methods

This study was approved by the Human Research Ethics Committees (Approval number: 2018001570) and Animal Ethics Committees (SVS/400/18) of The University of Queensland.

### 2.1. Equipment

The custom design and build of an ambulatory datalogger and sensor unit was commissioned for this project from RobacScience Australia (Blue Mountains, New South Wales, Australia). The unit was designed with a metal user handle for a human on one end of an ‘S’ geometry load cell (Meltrons Australia MT501) with a load capacity of 100 kg. The opposing end of the load cell has an M8 stainless steel eyebolt to allow a simple connection with a l.4-m commercial dog leash (Rogz Snake Leash) with minimal inherent stretch to avoid the load cell under reading and therefore cause tension errors. The signal of the load cell was amplified and digitalised with an HX711 load cell amplifier/analog-to-digital-converter.

The triaxial accelerometer component of a Bosch BNO055 nine axis MEMS (microelectromechanical system) inertial measurement device, was setup to operate in “linear accelerometer “mode. For this application, the direction of acceleration (sign of the acceleration vector) was the required data, rather than the absolute value. The gravity component of acceleration was filtered out. The system sampling frequency was determined by matching the accelerometer with the load cell at 10 Hz, allowing load cell tension and acceleration in X, Y, and Z directions to be synchronously recorded. It was recognised that leash orientation would change with size of the dog and human handler, as well as the rotation of the leash tension device. The software combines X, Y, Z axis accelerometry data with the scalar load tension to output a vector (i.e., load pulled in a direction). In total, the leash tension meter weighed around 720 g.

### 2.2. Software

A Windows 10 personal computer (PC) program for data collection and real-time display was written in C#. Data were received from the canine leash tension meter via an ESP32 receiver microcontroller connected to a serial port. The PC software displayed both the raw tension meter data and accelerometer data on two real-time line plots. Finally, the raw data were written to a timestamped CSV (comma-separated values) format file.

### 2.3. Equipment Validation

The load cell was tested with a series of static loads with different weights in the vertical direction, and the gradient, offset and linearity of the cell were found. A load resolution of 100 g was achievable over the full range. The Bosch BNO055 accelerometer was factory-calibrated and its functionality was tested by shaking the accelerometer to ensure that the onboard accelerometer properly responded to outside forces. In addition, the meter included an ESP32 microcontroller (Expressif Systems, Shanghai, China) for datalogging and transmitter communication functions (Wi-Fi/Bluetooth), user interface buttons, LEDs (light-emitting diodes), and a USB (universal serial bus) lithium cell charger/regulator.

### 2.4. Measurement of Body

Body size–weight represents the interaction of body height (cm), body length (cm), body weight (kg), and body condition score (BCS). Body height was measured from the ground to the dorsal scapular rim and body length was measured from the cranial aspect of the shoulder joint to the caudal aspect of the sciatic tuberosity [12]. Body weight was measured by RSPCA staff regularly. A 9-point scale BCS system was used to measure the BCS of dogs [13].

### 2.5. Field Deployment

Validation was conducted at the Royal Society for the Prevention of Cruelty to Animals, Queensland (RSPCA, Deakin, QLD, Australia), using 111 shelter dogs and 74 volunteers. Dogs were categorized by RSPCA staff into different levels: 1, 2, 3, and 3 + based on their behaviour during their daily walk. Level 1 dogs walked on a loose leash most of the time. Level 2 dogs pulled the leash during the walk occasionally and had more undesirable behaviours than level 1 dogs. Level 3 dogs tended to pull the leash fiercely due to excitement or timidity. Level 3 + dogs had severe behavioural issues, such as overt aggressiveness or fearfulness; however, they did not necessarily pull more than level 3 dogs. Behaviours on walks were daily monitored by shelter staff and volunteers, and levels may have changed accordingly. Dogs classified at the different levels were matched to volunteers of the right experience and training level for their daily walk.

**Figure 1:**
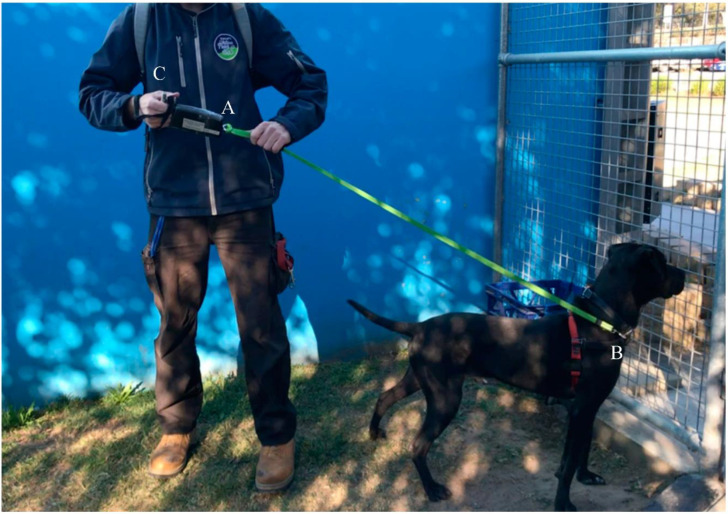
Demonstration of the canine leash tension meter. A l.4-metre-long leash was connected to the tension meter (**A**) and then was attached to the collar and harness in front of the dog’s chest (**B**). The handler held the metal handle on the other end of the tension meter (**C**).

**Figure 2:**
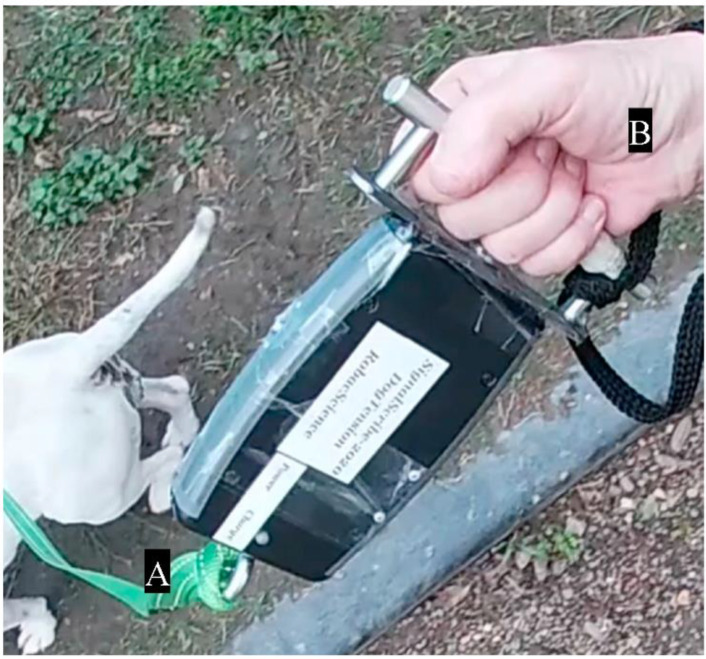
Close-up view of the canine leash tension meter. A l.4-metre-long leash was connected to the tension meter through the stainless-steel eyebolt (**A**) and the handler held the metal handle on the other end of the tension meter (**B**).

Walking level was assessed based on the dog’s walking, from leaving and returning to the kennel, including areas with more stimuli presented. However, to standardise the experimental procedure, leash tension was only recorded when dogs were on the designated pathway away from distractions at the shelter. Before every walk, the researcher held the leash tension meter vertically downward for 10 s without connecting to the dog. The signals generated were later used to help calibrate the recorded data using MATLAB (MATLAB^®^ and Statistics Toolbox Release 2018 b, The MathWorks, Inc., Natick, Massachusetts, MA, USA). All dogs at the RSPCA wore a plain neck collar and a chest harness. One end of the leash was connected to the stainless-steel eyebolt of the leash tension meter (Figure 1 and Figure 2) while the other end was attached to both the collar and the harness in front of the dog’s chest (Figure 1) in order to prevent them from accidentally escaping during the walk. The leash was attached to the front of the chest so as to provide a better control over the dog when it was lunging [14].

A laptop (Swift 3, Acer Inc., New Taipei City, Taiwan) was carried by the volunteer in a backpack for data collection, and a camera (GoPro Hero 7 Silver, GoPro^®^, California, CA, USA) was mounted on the volunteer’s head to record the interaction. Before walking, the volunteer was directed to pull the two ends of the device and hold the pulling for 3 s by counting slowly “1, 2, 3”. This procedure was repeated three times in order to synchronize the tension data with the video. During the walk, the volunteer was instructed not to touch the leash unless the dog got tangled.

### 2.6. Data Processing

Leash tension and pulling directions were calculated using MATLAB^®^ (MATLAB^®^and Statistics Toolbox Release 2018 b, The MathWorks, Inc., Natick, Massachusetts, MA, USA). The start and end of each file were determined by matching the timestamps of video and the leash tension file; also by matching three signal peaks at the beginning of the walk with the three repeated “1, 2, 3” verbal cues counted by the volunteer. Data were interpolated in order to make the sample times evenly spaced. Tension recordings were tared by deducting the minimal value, which visually equals the baseline value when the device is not connected to the dog. Peak and average tension over the walk were calculated.

A ‘pull event’ was defined as a sharp peak of tension greater than the baseline tension, which corresponded to a sudden burst of pulling initiated by either the dog, the handler or both at the same time. In order to investigate ‘pull events’, a moving average filter was used to remove the baseline tension from the signal. A peak identification algorithm was then used to determine when ‘pulling events’ occurred, with 0.1% of the body weight force set as a threshold. An event started when the filtered tension exceeded the threshold and ended when either the filtered tension returned to below the threshold or the sign of the filtered tension gradient changed from negative to positive (indicating the start of a new pull event). In addition, the directional signal of the accelerometer during the sample immediately prior to the start of a ‘pull event’ was used to determine the pulling direction (who initiated the pull: dog or human). Once the pull events were determined, the baseline tension was added back into the signal.

Net maximal tension (NTmax), maximal tension by dog (DTmax) and handler (HTmax) were defined as the maximal tension throughout the walk, recorded for the dog and handler, respectively. Mean tension was calculated by averaging all tension peaks above the threshold. Net mean tension (NTmean), mean tension by dog (DTmean), and mean tension by handler (HTmean) were defined as the mean tension throughout the walk, recorded for the dog and handler, respectively. Dog pulling frequency (DPF) and handler pulling frequency (HPF) were calculated by dividing the number of pulling events caused by the dog and the handler, respectively, by the total walking time.

### 2.7. Statistical Analysis

The validation process formed part of a larger research project that explores the behavioural interactions between shelter dogs and volunteer walkers. To better understand how humans and dogs affect the leash tension, human demographics, human personality, canine demographics and the canine behavioural assessment results were obtained. This paper focuses on validating the new method by investigating tension parameters at different dog body sizes (body height and length), weight (kg), ages, and ease of walking.

Statistical analysis was conducted using RStudio Version 1.2.1335. Generalised linear mixed models were constructed to analyse the relationships between independent variables and leash tension. To reduce the numbers of independent variables, a bivariate generalized linear model was first used to analyse each combination of dependent (leash tension and pulling frequency) and independent (human and dog demographics, human personality, canine behavioural assessment) variables. Independent variables with *p* values < 0.2 and all independent variables that were logically expected to have an effect on the dependent variable, regardless of the *p*-value, were included in the generalized linear mixed model. Regression analysis began with a full model, in which all candidate variables were defined as predictors of interest (fixed effects). Participants’ ID number and dogs’ ID number were entered as random effects. Variables with the least significant *p*-values were then removed in a stepwise manner until the results of the model became consistent. In addition to assessing significance, the change in the Bayesian Information Criteria (BIC) was used to assess whether the model improved by entering or removing variables. To meet the assumption of normal distribution of residuals, log 10 transformations were conducted on dependent variables (maximal and mean tension and pulling frequency). The assumption of normal distribution of residuals was assessed by observation of quantile–quantile plots for all analyses. The assumption of homogeneity of variance of residuals was confirmed with either Levene’s Test or visual inspection of boxplots. The assumption of no collinearity between covariates was evaluated with variance inflation factors (VIF < 2) [15].

## 3. Results

The mean age of dogs was 44.82 (± 29.37) months old and the mean weight was 24.43 (± 6.65) kg. The mean body height was 52.04 (± 6.29) cm and the mean body length was 56.05 (± 5.93) cm. The mean body condition score was 4.59 (± 1.07). This research recorded 370 on-leash walks, including 13 (3.51%) walks involving level 1 dogs, 162 (43.78%) involving level 2 dogs, 184 (49.73%) involving level 3 dogs, and 11 (2.97%) involving level 3 + dogs. The first hypothesis regarding the relationship between body size–weight and the leash tension and pulling frequency was supported, as there were significant effects of dog size and body weight on leash tension. Dog size and body weight were positively correlated with log 10 of NTmax (*p* < 0.001), DTmax (*p* = 0.001), DTmean (*p* = 0.0034), and HTmean (*p* = 0.028), but negatively correlated with log 10 of DPF (*p* = 0.018) and HPF (*p* = 0.001). The age of dog was negatively associated with log 10 of DPF (*p* = 0.0034) and HPF (*p* = 0.027), which supported the second hypothesis. The third hypothesis was partially met. The behavioural level of dogs was not significantly correlated with the leash tension contributed by human handlers, and also there was no difference between level 1 and level 2 or 3 dogs in terms of the leash tension. However, compared with level 3 dogs, level 2 dogs had significantly lower log 10 of NTmax (*p* = 0.014), DTmax (*p* = 0.036), and DTmean (*p* = 0.021) (Table 1).

## 4. Discussion

Rein tension meters have been used to quantify the human–horse dyads in equestrianism, while in previous canine research, associations between leash tension and potential health concerns in dogs have been only investigated by pulling the leash with fixed strengths [1,2]. The newly developed canine tension meter provides an accurate real-time measurement of forces exerted on the dog’s leash. Unlike previous equine rein tension meters, the canine meter differentiates between contributors of the force by correlating force with direction.

Larger and heavier dogs are generally stronger as they have more muscle mass, hence they had greater leash tension, which supported the first hypothesis. Moreover, since larger dogs are generally more cooperative [16], the expected negative correlation between dog size and pulling frequency was observed. This was mirrored on the human side as handlers needed stronger and steadier control over larger dogs.

With respect to the second hypothesis of dog age, young dogs are generally more active but less predictable [11], therefore, pulling more frequently. This hypothesis is also supported by the negative correlation between dog age and pulling frequency. In response to the dog’s behaviour, handlers also tended to correct the dog by tugging on the leash more often.

The ease of walking level influenced the pulling by dogs but not the pulling by handlers, indicating that the handler was less sensitive to this level of variation in canine pulling. No significant difference was observed in terms of leash tension between level 1 and level 3 dogs probably due to the small sample size of walks involving level 1 dogs (*n* = 13/370). Another possible explanation may be that the ease of walking level given by RSPCA staff was not as accurate and objective as the leash tension meter. However, in line with our third hypothesis, maximal and mean leash tension were lower in dogs rated as better behaved on walks (level 2 versus 3), suggesting that the leash tension meter could be an objective tool for grading canine behaviour when walking on a leash. This could be used to improve both the handler’s and dog’s experiences by matching dogs to levels of handler experience.

Walking level was determined by the dog’s behaviour during the entire walk (i.e., from leaving to returning to the kennel, which included areas with stimuli such as people and other dogs). However, to standardise the experimental procedure, leash tension was only recorded when dogs were in the designated pathway away from stimuli. Therefore, it is expected that the presence of conspecific or environmental challenges would result in greater differences in leash tension among dogs of different behavioural levels. Consequently, handlers may be more likely to detect the tension differences.

A limitation of the version of device used in the results presented here was found to be the printed circuit board (PCB) antenna. In certain orientations between the PC and the leash tension meters’ PCB antenna, two large metal components, the load cell and the user handle, could shield radio frequency resulting in up to 20% data loss. The device has subsequently been upgraded with an external antenna and SD card for simultaneous SD card and live telemetry records.

## 5. Conclusions

The canine leash tension meter is a robust and valid approach in exploring the real-world scenario of human–dog interactions when walking on a leash. It measures the leash tension and, most importantly, detects the direction of pulling. Larger and heavier dogs resulted in higher leash tension but lower pulling frequency at both the dog and the handler ends. More frequent pulls were observed for younger dogs, and a similar pattern was observed at the human end. Dogs considered better behaved during a walk, based on the RSPCA walking level, created lower leash tension but this was not observed for human handler behaviour. It is unclear how strong the force and how long the force exerted over time may compromise the health of dogs. In equine science, peak saddle pressures > 4.67 kPa can cause damage through lack of tissue perfusion, and pressures > 30 kPa under a saddle are associated with back pain [17]. Further investigation into canine leash tension meter could involve fitting it with a pressure sensor [2] to explore the effects of different restraint types (e.g., neck collar, head collar, and different types of harness) on the leash tension and pressure on the body of dogs. Additionally, the tension meter may facilitate longitudinal and prospective research on the continual effects of a tight leash on the welfare and health of dogs.

In future applications, the canine leash tension meter can be used to improve training and encourage the use of empathetic and reward-based training techniques. A broader applicability may be in veterinary behavioural medicine, to evaluate the severity of leash reactivity in dogs and monitor the improvement following training or treatment [18].

## Figures and Tables

**Table 1:** Generalized linear mixed model of effects of dog size–weight, dog age, and dog walking levels on leash tension and pulling frequency (dependent variables).

	Log _10_ NT_max_	Log _10_ NT_mean_	Log _10_ DT_max_	Log _10_ DT_mean_	Log _10_ DPF ^1^	Log _10_ HT_max_	Log _10_ HT_mean_	Log _10_ HPF ^1^
Dog size ^2^	*β* 6.0 × 10^−7^ SE 2.0 × 10^−7^ *p* < 0.001	*β* 1.0 × 10^−7^ SE 1.0 × 10^−7^ *p* 0.27	*β* 6.2 × 10^−7^ SE 2.0 × 10^−7^ *p 0.001*	*β* 3.8 × 10^−7^ SE 1.3 × 10^−7^ *p 0.0034*	*β* −1.0 × 10^−6^ SE 3 × 10^−7^ *p 0.018*	*β* 2.3 × 10^−7^ SE 2 × 10^−7^ *p* 0.20	*β* 2.9 × 10^−7^ SE 1.3 × 10^−7^ *p 0.028*	*β −9.0 × 10^−7^* *SE 3 × 10^−7^* *p 0.001*
Dog age	*β* −1.0 × 10^−3^ SE 1.3 × 10^−3^ *p* 0.44	*β* −2.9 × 10^−4^ SE 9.2 × 10^−4^ *p* 0.75	*β* –9.2 × 10^−4^ SE 1.5 × 10^−3^ *p* 0.54	*β* 3.5 × 10^−4^ SE 1.0 × 10^−3^ *p* 0.97	*β* –6.7 × 10^−3^ SE 2.3 × 10^−3^ *p 0.0034*	*β* −5.8 × 10^−4^ SE 1.4 × 10^−3^ *p* 0.68	*β* −7.4 × 10^−5^ SE 1.0 × 10^−3^ *p* 0.94	*β* −*4.7 × 10^−3^* *SE 2.1 × 10^−3^* *p 0.027*
Walking Level ^3^								
Level 1	μ, SD 2.76, 1.09 *β* −0.032 SE 0.17 *p* 0.85	μ, SD 0.51, 0.22 *β* −0.041 SE 0.13 *p* 0.74	μ, SD 2.61, 1.56 *β* −0.021 SE 0.18 *p* 0.91	μ, SD 1.03, 0.36 *β* 0.067 SE 0.12 *p* 0.59	μ, SD 0.08, 0.05 *β* −0.22 SE 0.27 *p* 0.41	μ, SD 2.28, 0.84 *β* −0.025 SE 0.18 *p* 0.89	μ, SD 0.94, 0.31 *β* −0.054 SE 0.13 *p* 0.67	μ, SD 0.09, 0.07 *β* −0.39 SE 0.25 *p* 0.11
Level 2	μ, SD 3.38, 1.9 *β* −0.17 SE 0.070 *p* 0.014	μ, SD 0.54, 0.23 *β* −0.11 SE 0.058 *p* 0.067	μ, SD 2.93, 1.67 *β* −0.16 SE 0.076 *p 0.036*	μ, SD 1.07, 0.46 β −0.12 SE 0.053 *p 0.021*	μ, SD 0.17, 0.14 *β* −0.0029 SE 0.11 *p* 0.98	μ, SD 2.75, 1.55 *β* −0.14 SE 0.074 *p* 0.056	μ, SD 1.04, 0.44 *β* −0.096 SE 0.053 *p* 0.072	μ, SD 0.17, 0.13 *β* −0.14 SE 0.10 *p* 0.16
Level 3	μ, SD 4, 2.01	μ, SD 0.62 0.26	μ, SD 3.53, 1.93	μ, SD 1.22, 0.51	μ, SD 0.21, 0.14	μ, SD 3.26, 1.82	μ, SD 1.22, 0.55	μ, SD 0.2, 0.13
Level 3+	μ, SD 4.6, 2.2 *β* 0.16 SE 0.16 *p* 0.33	μ, SD 0.73, 0.25 *β* 0.11 SE 0.14 *p* 0.43	μ, SD 3.87, 1.5 *β* 0.16 SE 0.17 *p* 0.36	μ, SD 1.44, 0.46 *β* 0.15 SE 0.12 *p* 0.22	μ, SD 0.19, 0.16 *β* 0.21 SE 0.24 *p* 0.38	μ, SD 3.99, 2.71 *β* 0.18 SE 0.17 *p* 0.28	μ, SD 1.31, 0.4 *β* 0.11 SE 0.12 *p* 0.38	μ, SD 0.14, 0.09 *β* 0.25 SE 0.22 *p* 0.27

All dependent variables were entered into generalized linear mixed model using log _10_ transformation. NT_max_: maximal net leash tension. NT_mean_: mean net leash tension. DT_max_: maximal leash tension caused by dog. DT_mean_: mean leash tension caused by dog. HT_max_: maximal leash tension caused by handler. HT_mean_: mean leash tension caused by handler. DPF: dog pulling frequency. HPF: handler pulling frequency. ^1^ Pulling frequency = (Numbers of pulls)/(walking duration). ^2^ Dog size is the interaction of the dog’s body height, body length, body weight, and body condition score (9-point scale) [13]. ^3^ Level 3 was used for comparison. μ: mean (kg force). SD: standard deviation of μ*. β*: regression coefficient. SE: standard error of *β. p*: *p* value of the model.
